# Activation of Host IRE1α-Dependent Signaling Axis Contributes the Intracellular Parasitism of *Brucella melitensis*

**DOI:** 10.3389/fcimb.2018.00103

**Published:** 2018-04-20

**Authors:** Aseem Pandey, Furong Lin, Ana L. Cabello, Luciana F. da Costa, Xuehuan Feng, Hui-Qiang Feng, Ming-Zhe Zhang, Takao Iwawaki, Allison Rice-Ficht, Thomas A. Ficht, Paul de Figueiredo, Qing-Ming Qin

**Affiliations:** ^1^Key Laboratory of Zoonosis Research, Ministry of Education, College of Plant Sciences, Jilin University, Changchun, China; ^2^Department of Microbial Pathogenesis and Immunology, Texas A&M Health Science Center, College Station, TX, United States; ^3^Department of Veterinary Pathobiology, College of Veterinary Medicine, Texas A&M University, College Station, TX, United States; ^4^Division of Cell Medicine, Department of Life Science, Medical Research Institute, Kanazawa Medical University, Uchinada, Japan; ^5^Department of Molecular and Cellular Medicine, Texas A&M Health Science Center, College Station, TX, United States; ^6^Norman Borlaug Center, Texas A&M University, College Station, TX, United States

**Keywords:** *Brucella melitensis*, unfolded protein response (UPR), inositol-requiring enzyme 1 (IRE1), ULK1, autophagy, intracellular trafficking and replication

## Abstract

*Brucella* spp. are intracellular vacuolar pathogens that causes brucellosis, a worldwide zoonosis of profound importance. We previously demonstrated that the activity of host unfolded protein response (UPR) sensor IRE1α (inositol-requiring enzyme 1) and ER-associated autophagy confer susceptibility to *Brucella melitensis* and *Brucella abortus* intracellular replication. However, the mechanism by which host IRE1α regulates the pathogen intracellular lifestyle remains elusive. In this study, by employing a diverse array of molecular approaches, including biochemical analyses, fluorescence microscopy imaging, and infection assays using primary cells derived from *Ern1* (encoding IRE1) conditional knockout mice, we address this gap in our understanding by demonstrating that a novel IRE1α to ULK1, an important component for autophagy initiation, signaling axis confers susceptibility to *Brucella* intracellular parasitism. Importantly, deletion or inactivation of key signaling components along this axis, including IRE1α, BAK/BAX, ASK1, and JNK as well as components of the host autophagy system ULK1, Atg9a, and Beclin 1, resulted in striking disruption of *Brucella* intracellular trafficking and replication. Host kinases in the IRE1α-ULK1 axis, including IRE1α, ASK1, JNK1, and/or AMPKα as well as ULK1, were also coordinately phosphorylated in an IRE1α-dependent fashion upon the pathogen infection. Taken together, our findings demonstrate that the IRE1α-ULK1 signaling axis is subverted by the bacterium to promote intracellular parasitism, and provide new insight into our understanding of the molecular mechanisms of intracellular lifestyle of *Brucella*.

## Introduction

Infectious diseases caused by bacterial pathogens contribute significantly to global disease burden. *Brucella* is one such animal pathogen that causes brucellosis, a worldwide zoonosis of profound importance (Pappas et al., 2006). Brucellosis has eluded systematic attempts at eradication for more than a century (Godfroid et al., 2002), and to date no approved human vaccine is available (Ficht and Adams, 2009; Pandey et al., 2016). These features contribute to the classification of *Brucella* as a potential bioterror agent and economic threat, and to the enormous interest expressed in this pathogen by the biosecurity and world health communities.

*Brucella* is an intracellular vacuolar pathogen that invades many cell and tissue types (de Figueiredo et al., 2015). Experiments over the last two decades have revealed the capacity of *Brucella* to evade intracellular destruction by restricting fusion of the *Brucella*–containing vacuoles (BCVs) with lysosomes. BCVs harboring internalized *Brucella* traffic from endocytic compartments to a replicative niche within vacuoles (rBCVs) that are decorated with markers of the endoplasmic reticulum (ER). BCVs can also accumulate LAMP-1 positive autophagic membranes that constitute a distinctive aspect of the intracellular lifestyle of the pathogen (Starr et al., 2012). The VirB type IV secretion system (T4SS) is a significant virulence factor that regulates *Brucella* intracellular trafficking (Marchesini et al., 2011; Paredes-Cervantes et al., 2011; Sá et al., 2012; Smith et al., 2012), and organisms that lack this system fail to establish an intracellular replicative niche.

The Unfolded Protein Response (UPR) is an evolutionarily conserved signaling pathway that mediates cellular adaptation to protein-folding stress in the ER (for review, see Gardner et al., 2013). The UPR signals through the stress sensors IRE1α, ATF6, and PERK located in the ER membrane. IRE1α plays a central role in initiating UPR by activating biochemically distinct pathways. In the X Box Binding Protein 1 (XBP1)-dependent pathway, an endonuclease activity in the cytoplasmic tail of IRE1α catalyzes the splicing of XBP1 mRNA. The spliced message is then translated to generate XBP1 transcription factor (Ron and Walter, 2007), which controls the expression of UPR genes that encode ER chaperones and other proteins that mitigate the harmful consequences of unfolded protein accumulation. In the XBP1-independent pathway, IRE1α activity on downstream cytosolic protein targets promotes apoptosis or induces autophagy (Levine and Kroemer, 2008), independently of XBP1 transcription factor activity (Wei et al., 2008). The XBP1-independent IRE1α pathway is activated by a complex of IRE1α-associated proteins that includes Apoptosis Signal-Regulating Kinase 1 (ASK1) (Ichijo et al., 1997; Nishitoh et al., 1998; Hatai et al., 2000), B cell lymphoma 2 (BCL2) Homologous Antagonist/Killer (BAK) and Bcl2 Associated X protein (BAX) (Wei et al., 2001; Hetz et al., 2006). The mechanisms by which this signaling complex promotes autophagosome biogenesis remain areas of investigation.

Our research group implicated the subversion of IRE1α, and ER-associated autophagy, as being critical to the intracellular lifestyle of *Brucella* (Qin et al., 2008). Starr and colleagues extended these findings by showing that *Brucella abortus* replication occurs in ER-like rBCVs, preceding the acquisition of autophagic markers and the formation of autophagic BCVs (aBCVs). aBCVs were also hypothesized to mediate the release of the pathogen from host cells (Starr et al., 2012). A role for UPR in regulating *Brucella* infection also emerged from work in which Smith et al. demonstrated that *B. melitensis* infection upregulated the expression of UPR target genes and induced XBP1 mRNA splicing in murine macrophages (Smith et al., 2013). Taguchi and colleagues recently showed that *B. abortus* infection activates host IRE1α, but not PERK or ATF6. Depletion of IRE1α dramatically reduced the intracellular replication of the bacterium (Taguchi et al., 2015). These data implicated UPR signaling pathways in conferring susceptibility to *Brucella* intracellular replication. However, the signaling cascades that control this process, and the cellular mechanisms regulated by these cascades, remained elusive. Here, we address this gap in our understanding by demonstrating that a novel IRE1α-ULK1 signaling axis contributes to conferring susceptibility to *Brucella* intracellular replication. In addition, we show that IRE1α-directed activation of components of the host autophagy program promotes the proper intracellular trafficking and replication of the agent.

## Materials and methods

### Cell culture

All the cells including murine macrophage J774.A1 cells, RAW264.7 macrophages, mouse bone marrow derived macrophages (BMDMs), murine embryonic fibroblasts (MEFs), and *Drosophila* S2 cells were cultured as previously described (Qin et al., 2008, 2011; Pandey et al., 2017).

### Bacterial strains and infection

*Brucella melitensis* WT strains 16M and 16M-GFP (Qin et al., 2008) were used in this study. Bacterial cultivation and host infection were performed using previously described methods (Qin et al., 2008). Host cells including *Drosophila* S2 cells, mouse MEFs. RAW264.7 macrophages and BMDMs, were infected with *B. melitensis* strain 16M (Bm16M) or 16-GFP at an MOI of 100, unless otherwise indicated. After centrifugation for 5 min at 200 × g, infected host cells were then incubated at 29°C (S2 cells) or 37°C (mammalian cells) for 30 min. Culture media were removed, and then the infected cells were rinsed with 1 × phosphate buffered saline (PBS, pH 7.2). Fresh media supplemented with 40 μg/ml gentamicin was then added for 30 min to kill extracellular bacteria. Infected cells were lysed for bacterial internalization assay or continuously incubated in this antibiotic for various lengths of time and performed CFU assay to assess the bacterial intracellular growth as previously described (Qin et al., 2008). *Brucella* infections, CFU assays, and lysate preparation of *Brucella*-infected host cells were performed in the BSL3 facilities located in the College of Veterinary Medicine at Texas A & M University.

### Drug treatments

Murine RAW264.7 macrophages or BMDMs were co-incubated in 24-well plates with assorted chemicals including SP600125 (Bennett et al., 2001), ethyl 2,7-dioxo-2,7-dihydro-3*H*-naphtho[1,2,3-*de*]quinoline-1-carboxylate (NQDI1) (Volynets et al., 2011), KIRA6 (Ghosh et al., 2014) at the indicated concentrations. Cells were treated with the drugs 1 h before or after, and during, infection with Bm16M strains. To evaluate Bm16M internalization, after 30 min of infection and three washes with 1 × PBS, fresh media supplemented with the same concentration of drugs and 40 μg/ml gentamicin was added to kill extracellular bacteria. After an additional 30 min of incubation, the cells were lysed and the CFU per well was determined as previously described (Qin et al., 2008). To assess *Brucella* intracellular replication, CFU analysis was performed at 24, 48, and 72 h.p.i. To investigate whether the tested compounds inhibit *Brucella* growth, the chemicals were individually added to *Brucella* TSB cultures at 37°C and incubated for 1, 24, 48, or 72 h. To evaluate the potential inhibitory effects of these tested drugs, Bm16M growth in the presence of these drugs was assessed using a CFU plating approach as previously described (Qin et al., 2008).

### dsRNA and lentivirus-mediated depletion of host proteins

dsRNA-mediated knockdown of target genes in *Drosophila* S2 was performed as previously described (Qin et al., 2008). For RAW264.7 macrophages, lentivirus mediated generation of stable target gene knockdown cell lines was performed as the previously described (Chaki et al., 2013; Pandey et al., 2017). Briefly, the pSuperRetro retrovirial vector system (OligoEngine, Inc.) was used to construct vectors to knockdown expression of target genes in murine RAW264.7 macrophages as per the manufacturer's instructions. The reported shRNAs (Alers et al., 2011; Pandey et al., 2017) were used for knockdown of the expression of ULK1 gene. RAW264.7 macrophages (2.0 × 10^5^) cultured in six-well plates were used for transfection, and puromycin was used for screening clones in which the insert was stably integrated. Western blot was performed to validate the depletion of the targeted proteins.

### BMDM harvest and cultivation

*LysM-Ern1*^mut/*mut*^ and control mice were generated under the auspices of approval by the Texas A & M University Institutional Animal Care and Use Committee in an Association for Assessment and Accreditation of Laboratory Animal Care International Accredited Animal Facility. BMDMs collected from the femurs of *Ern1*^mut/mut^ and control littermates were cultivated in L929-cell conditioned media [DMEM medium containing 20% L929 cell supernatant, 10% (v/v) FBS, penicillin (100 U/ml) and streptomycin (100 U/ml)]. After 3 days of culture, non-adherent precursors were washed away and the retained cells were propagated in fresh L929-cell conditioned media for another 4 days. BMDMs were split in 24-well plates (2.5 × 10^5^ cells/well) in L929-cell conditioned media and cultured at 37°C with 5% CO_2_ overnight before use.

### Viability and membrane permeability assays of infected host cells

Host cells coincubated with or without various drugs and/or infected with *Brucella* were subjected to 0.2% trypan blue vital stain analysis at various time points post-treatment or -infection as previously described (Qin et al., 2008) to quantify viability or evaluate membrane permeability of drug-treated or Bm16M infected host cells. Host cells in which drug treatment or *Brucella* infection induced no significant differences in viability and membrane permeability were used in the experiments reported in this work.

### Fluorescence microscopy and immunofluorescence microscopy assay (IFMA)

To visualize Bm16M intracellular trafficking, the indicated host cells (5.0 × 10^4^) were seeded on 12-mm coverslips placed on the bottom of wells of 24-well plates and infected with Bm16M-GFP or Bm16M for various lengths of time. To analyze infections of less than 0.5 h, the infected cells were rinsed with 1 × PBS and then fixed with 3.7% formaldehyde at 4°C for overnight before immunofluorescence microscopy analysis (IFMA). Otherwise, at 0.5 h.p.i., the infected cells were washed with 1 × PBS and fresh media supplemented with 40 μg/ml gentamicin was added to kill extracellular bacteria. At the indicated time points post-infection, the infected cells were fixed and IFMA was performed as previously described (Qin et al., 2008, 2011; Pandey et al., 2017). The primary antibodies used were as follows: goat-anti *Brucella*, rabbit anti-LAMP-1; rabbit anti-cathepsin D; rabbit anti-mouse LC3; rabbit anti-Calreticulin; rabbit anti-ULK1, rabbit anti-Beclin 1 (Santa Cruz Biotech., Inc, 1:200-500). Samples were stained with Alexa Fluor 488-conjugated and/or Alexa Fluor 594-conjugated secondary antibody (Invitrogen/Molecular Probes, 1:1,000). Acquisition of confocal images, and image processing and analyses were performed as previously described (Qin et al., 2008, 2011; Pandey et al., 2017).

### Immunoblotting analysis

Protein sample preparation and Western immunoblotting were performed as previously described (Qin et al., 2011; Pandey et al., 2017). Primary antibodies used in the immunoblotting analysis included anti-p-ULK1, anti-ULK1, anti-ASK1, anti-p-ASK1, anti-SAPK/JNK, anti-p-SAPK/JNK (Cell signaling); anti-AMPKα, anti-p-AMPKα, anti-Atg9a (Thermo Scientific); anti-LC3, anti-p-ASK1 and anti-GAPDH (Santa Cruz Biotech., Inc); anti-BAK, anti-BAX (EMD Millipore), anti-p-IRE1α (GeneTex, Inc), anti-IRE1α (Novus Biologicals). Dilution of primary antibodies was 1:1,000. Secondary antibody HRP anti-IgG (Sigma-Aldrich, USA, 1:1,000~5,000) was used in the immunoblotting analysis. Densitometry of blots was performed using the ImageJ software package (http://rsbweb.nih.gov/ij/). The relative expression levels of target proteins and the ratio of blot LC3-II/LC3-I at the indicated time points were calculated as previously described (Qin et al., 2011). All Westerns were performed in triplicate and representative images are shown.

### Statistical analysis

All quantitative data were derived from results obtained in triplicate wells for at least three independent experiments. All the analyzed data were normalized with internal controls before preforming the Student's *t*-test to assess statistical significance between two experimental data sets or a one-way ANOVA test to evaluate the statistical differences of multiple comparisons of the data sets.

## Results

### IRE1α signaling contributes to conferring susceptibility to *B. melitensis* intracellular parasitism

We previously exploited a *Drosophila melanogaster* S2 cell system and RNAi technology to demonstrate that host IRE1α activity confers susceptibility to *B. melitensis* strain 16M (Bm16M) infection of the insect cells (Qin et al., 2008). To further dissect the molecular mechanisms by which host IRE1α controls the intracellular lifestyle of *Brucella*, we employed this convenient and established system to determine whether other UPR-related components regulate *Brucella* entry or replication in host cells. We found that XBP1 depletion did not affect the uptake or intracellular replication of the pathogen (Figure S1A). In addition, we observed that Bm16M replicated inefficiently (compared to controls) in S2 cells that had been individually depleted of key components in the XBP1-independent kinase cascade, including JAB1, ASK1, and JNK (Figure S1A). We also tested whether several host autophagy-related components, including Atg1 (ULK1), Atg2, Atg18 (WIPI1), Atg9 and Beclin 1, conferred susceptibility to Bm16M infection via CFU assay and image-based host cell infection analysis (Qin et al., 2008). We found that RNAi-mediated depletion of these proteins also reduced *Brucella* intracellular replication (Figure S1), thereby raising the possibility that an axis linking IRE1α and downstream autophagy proteins controls Bm16M intracellular parasitism.

### IRE1α activity controls Bm16M intracellular replication

**Figure 1:**
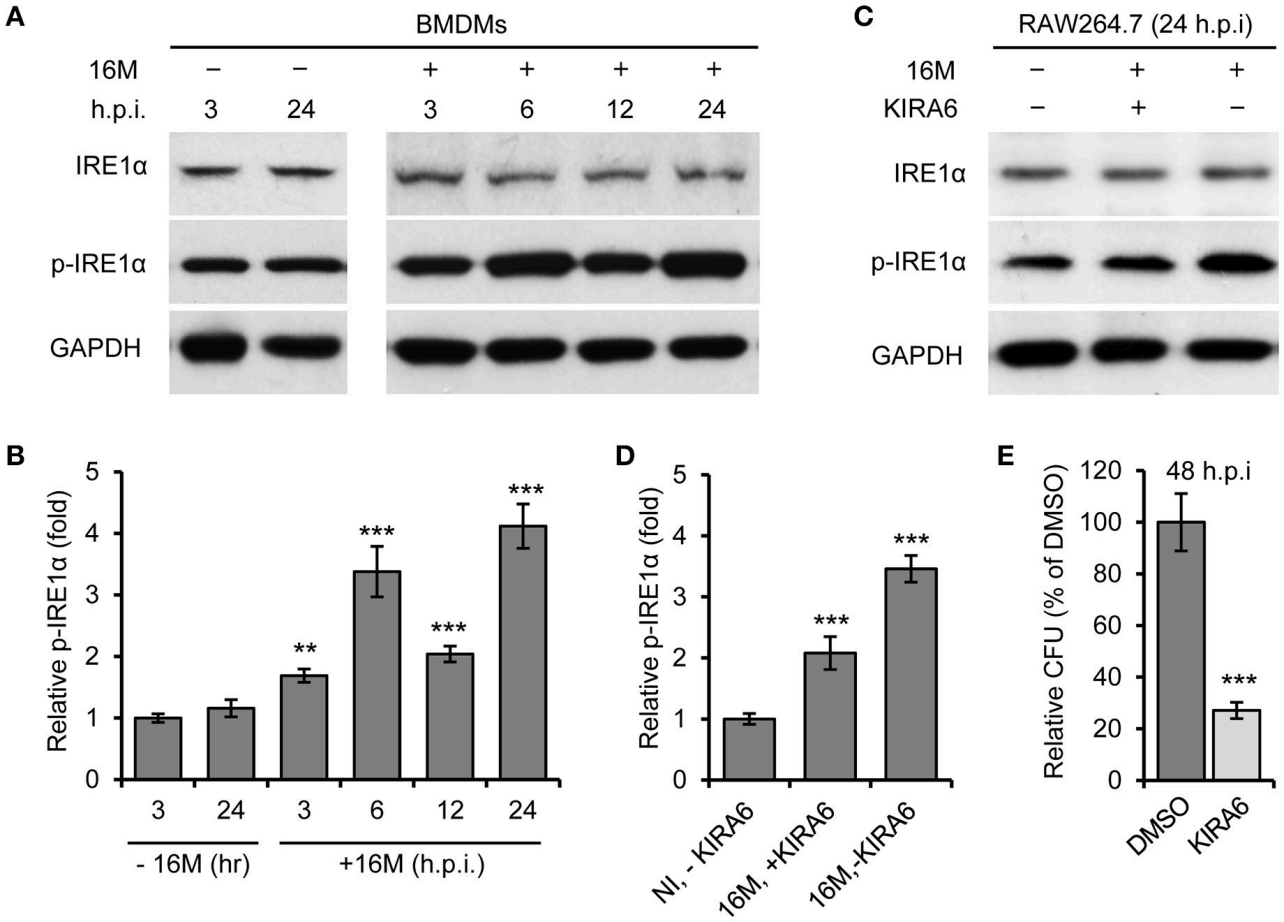
Activation of IRE1α contributes to Bm16M intracellular replication. Host cells were infected with Bm16M for various length of time. Samples were lysed for Western blot with the indicated antibodies or for CFU assays. **(A,B)** Bm16M infection activates host IRE1α in BMDMs **(A)** and quantification of the relative p-IRE1α level (mean of three blots for each time point) **(B)**. **(C,D)** Inhibition of IRE1α activity with its specific inhibitor KIRA6 reduced IRE1α activation during Bm16M infection **(C)** and quantification of the relative p-IRE1α level **(D)**. NI, no infection. **(E)** The intracellular replication of Bm16M was analyzed by gentamicin protection analysis in RAW264.7 macrophages treated with KIRA six before and during infection. Data represent means ± standard deviation (*SD*) from three independent experiments with triplicate wells examined for each treatment. ^**^p < 0.01 and ^***^p < 0.001, respectively.

We previously demonstrated that host factor IRE1α (ERN1) is required for Bm16M intracellular replication in mammalian cells (Qin et al., 2008). To test the hypothesis that Bm16M infection of host cells activates IRE1α activity, we monitored the activation of IRE1α during infection. We observed that the expression level of IRE1α was relatively unchanged during a time course (24 h) of infection in both BMDMs (Figure 1A) and RAW264.7 macrophages (Figure 1C). However, we found that IRE1α phosphorylation was enhanced over the same time period in BMDMs and RAW264.7 cells that had been infected with Bm16M (Figures 1A–D). The finding is consistent with the previous findings of host IRE1α activation by infection with *B. abortus* (Taguchi et al., 2015; Liu et al., 2016) or *B. suis* (Wang et al., 2016). As expected, IRE1α phosphorylation was also reduced in RAW264.7 cells treated with KIRA6, a specific inhibitor of IRE1α kinase activity (Ghosh et al., 2014), before and during Bm16M infection (Figures 1C,D). To assess the influence of host IRE1α activity on the intracellular replication of the pathogen, we used gentamicin protection assay (Qin et al., 2008) to examine the pathogen replication in IRE1α^+/+^ and IRE1α^−/−^ MEFs or in RAW264.7 macrophages treated with KIRA6, we found that MEFs derived from IRE1α ^−/−^ mice or RAW264.7 macrophages treated with the IRE1α kinase inhibitor also displayed lower levels of Bm16M intracellular replication than controls (Figure 1E, and data not shown). Therefore, the data supported the hypothesis that activation of IRE1α contributes to Bm16M intracellular parasitism.

### An activated IRE1α-to-JNK signaling cascade controls the intracellular replication and trafficking of Bm16M

**Figure 2:**
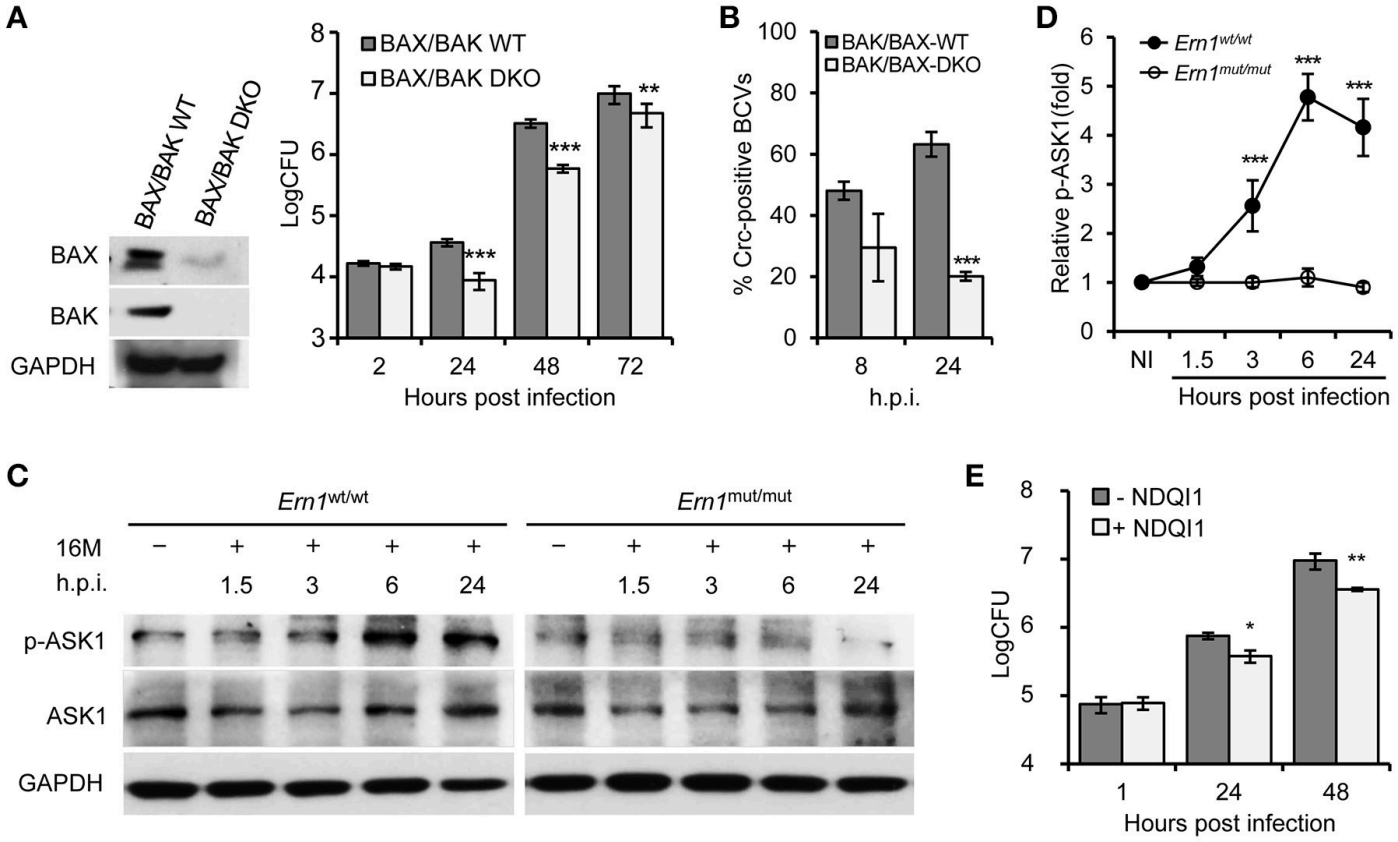
IRE1α downstream signaling components BAX/BAK and ASK1 contribute to Bm16M intracellular parasitism. The indicated drug-treated or untreated host cells were infected with Bm16M. At the indicated h.p.i., infected host cells were lysed for CFU or Western blot analysis. **(A)** The intracellular replication (right panel) was measured in MEFs harboring double deletion of host BAX and BAK genes (BAX/BAK DKO MEFs) as demonstrated in the left panel. **(B)** Quantification of Bm16M surrounded by the ER marker calreticulin (Crc-positive BCVs) in control and BAK/BAX DKO MEFs at the indicated time points post-infection. **(C,D)** IRE1α-dependent activation of host ASK1 during Bm16M infection of *Ern1*^mut/mut^ and *Ern1*^wt/wt^ BMDMs **(C)** and quantification of the relative p-ASK1 level (mean of three blots for each time point) **(D)**. NI, no infection. **(E)** Bm16M intracellular replication was measured in cells where ASK1 activity was inhibited by supplementation with NDQI1, a selective inhibitor of ASK1 kinase activity. Data represent means ± *SD* from three independent experiments with triplicate wells examined for each treatment. ^*^p < 0.05, ^**^p < 0.01, and ^***^p < 0.001, respectively.

To test the hypothesis that the activity of an IRE1α-activated signaling cascade contributes to the susceptibility of host cells to Bm16M intracellular parasitism, we investigated whether the intracellular replication or trafficking of the pathogen was altered in host cells that harbored deletions in several genes in this cascade, including BAX/BAK, ASK1, and JNK1, that were shown to be important in host cells alleviating UPR (Hetz et al., 2006), and in the S2-*Brucella* interaction system (Figure S1). Gentamicin protection assays revealed that MEF cells harboring double deletions in BAK/BAX (BAK/BAX DKO) reduced Bm16M intracellular replication compared to controls (Figure 2A). Bm16M also showed reduced trafficking to a calreticulin-positive replicative niche in these cells (Figure 2B). To better evaluate signaling events during Bm16M infection, we determined IRE1α-dependent activation of kinases (including ASK1, JNK1/2, etc.) in the IRE1α-activated signaling cascade in Bm16M-infected mouse *Ern1*^wt/wt^ and *Ern1*^mut/mut^ BMDMs derived from LysM-*Ern1*^mut/mut^ mice in which exons 21–22 of the *Ern1* gene encoding IRE1α were deleted from monocytes and macrophages (Iwawaki et al., 2009). Our data demonstrated that during the pathogen infection of mouse *Ern1*^wt/wt^ and *Ern1*^mut/mut^ BMDMs, ASK1 phosphorylation (Thr845) level increased over the same time period (24 h) in the infected *Ern1*^wt/wt^ BMDMs, indicating that IRE1α activity contributes to the infection-dependent phosphorylation of ASK1 (Figures 2C,D). Gentamicin protection assays indicated that host cells (RAW264.7 macrophages) pre-treated with NDQI1, a selective inhibitor of ASK1 kinase activity (Volynets et al., 2011), reduced *Brucella* intracellular replication (Figure 2E). These data indicated that host BAK/BAX and ASK1 activity support Bm16M intracellular replication.

**Figure 3:**
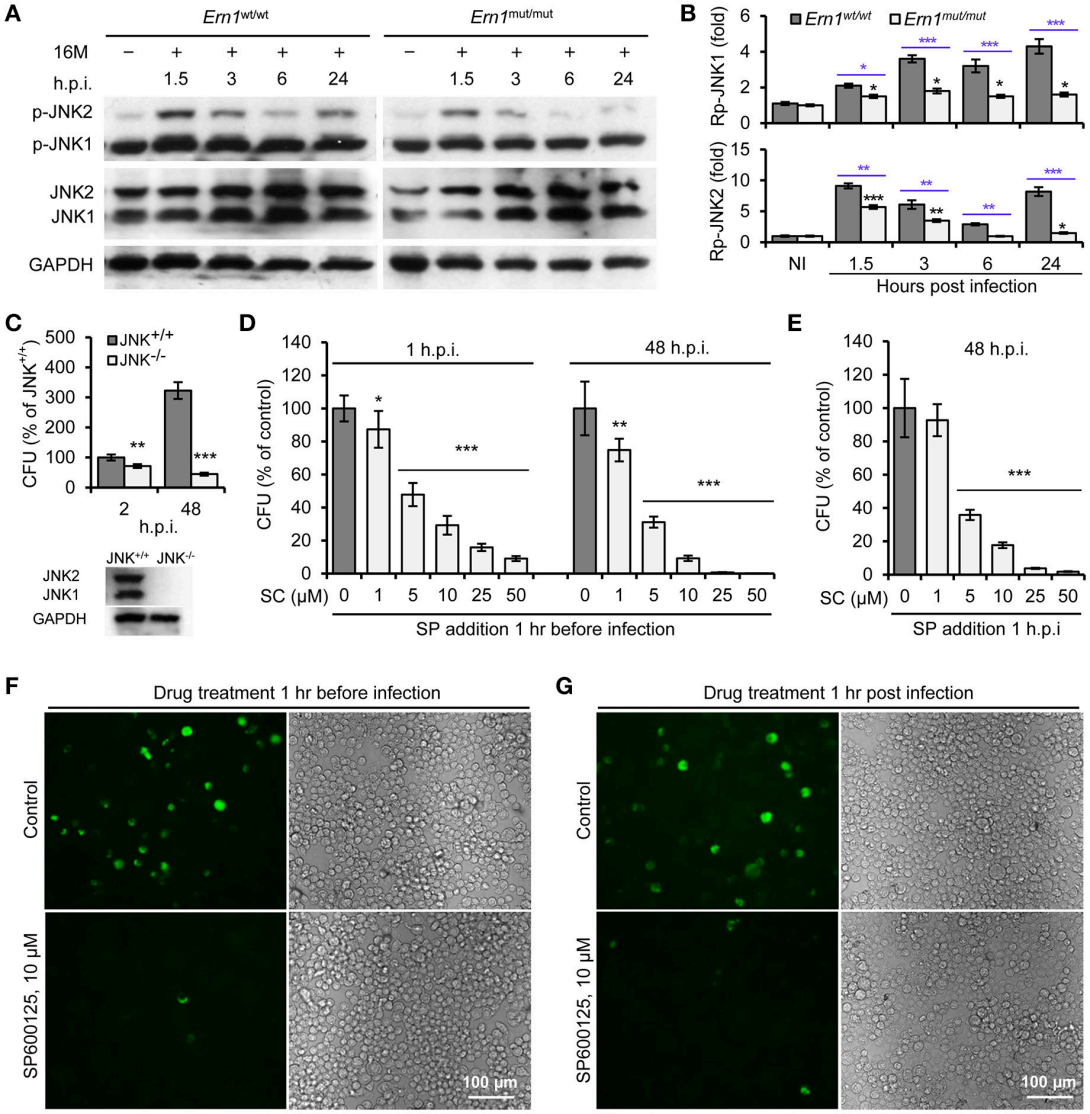
Activation of host JNK1/2 in IRE1α signaling cascade confers Bm16M infection. The indicated drug-treated or untreated host cells were infected with Bm16M. At the indicated h.p.i., infected host cells were lysed for Western blot or CFU analysis. **(A,B)** Activation of host JNK1/2 is IRE1α-dependent during Bm16M infection of *Ern1*^mut/mut^ and *Ern1*^wt/wt^ BMDMs **(A)** and quantification of the relative p-JNK1 (**B**, upper panel) and p-JNK2 **(B**, lower panel) levels (mean of three blots for each time point). Back and blue asterisks: significances when compared to the control of no infection of *Ern1*^*mut*/*mut*^ cells and to the infected *Ern1*^*mut*/*mut*^ cells at the same time points, respectively. **(C)** Bm16M infection (upper panel) was measured in host cells in which host JNK was ablated (lower panel, MEFs). **(D)** Determination of Bm16M entry into (left panel, 1 h.p.i.) and replication (right panel, 48 h.p.i.) in murine J774.A1 macrophages in which JNK activity was inactivated by pre-treatment with SP600125. **(E)** Inhibition of JNK activity reduced Bm16M intracellular replication in J774.A1 macrophages. **(F,G)** Representative images demonstrating the effects of inhibition of JNK activity in J774.A1 macrophages by SP600125 before **(F)** or after **(G)** Bm16M entry into host cells on intracellular replication of the pathogen at 48 h.p.i. NI, no infection. Rp-JNK1 and Rp-JNK2: relative phosphorylation levels of p-JNK1 and p-JNK2, respectively. SC: SP600125 concentration (μM); SP: SP600125. Data represent means ± *SD* from three independent experiments with triplicate wells examined for each treatment. ^*^p < 0.05, ^**^p < 0.01, and ^***^p < 0.001, respectively.

To determine IRE1α-mediated activity of JNK1/2 in Bm16M infection, we monitored phosphorylation (T183/Y185) of JNK1/2 during Bm16M infection of *Ern1*^wt/wt^ and *Ern1*^mut/mut^ BMDMs. We also determined the roles of JNK1/2 in Bm16M intracellular parasitism via gentamicin protection assays of the pathogen infection of mouse MEFs harboring JNK deletion or J774.A1 macrophages pre-treated with SP600125, a selective inhibitor of JNK kinase activity (Bennett et al., 2001). We found that Bm16M infection activated JNK1/2 in macrophages (Figures 3A,B) and MEFs (Figure S2), and that JNK1/2 displayed less infection-dependent phosphorylation in *Ern1*^mut/mut^ BMDMs than in corresponding controls (Figures 3A,B). Moreover, JNK1^−/−^ MEFs or J774.A1 macrophages pre-treated with SP600125 showed striking reductions in Bm16M infection compared to controls (Figures 3C,D,F). Consistent with these findings was the observation that when SP600125 was added to host cells after infection was initiated, reductions in Bm16M replication were also observed (Figures 3E,G). These data revealed that JNK1 activity plays a sustained role in enhancing susceptibility to intracellular replication by the pathogen. Taken together, these findings indicate that host IRE1α-to-JNK signaling contributes to the biogenesis of rBCVs as well as the intracellular replication of the pathogen.

### IRE1α-mediated activation of host Atg protein kinase ULK1 confers infection susceptibility

**Figure 4:**
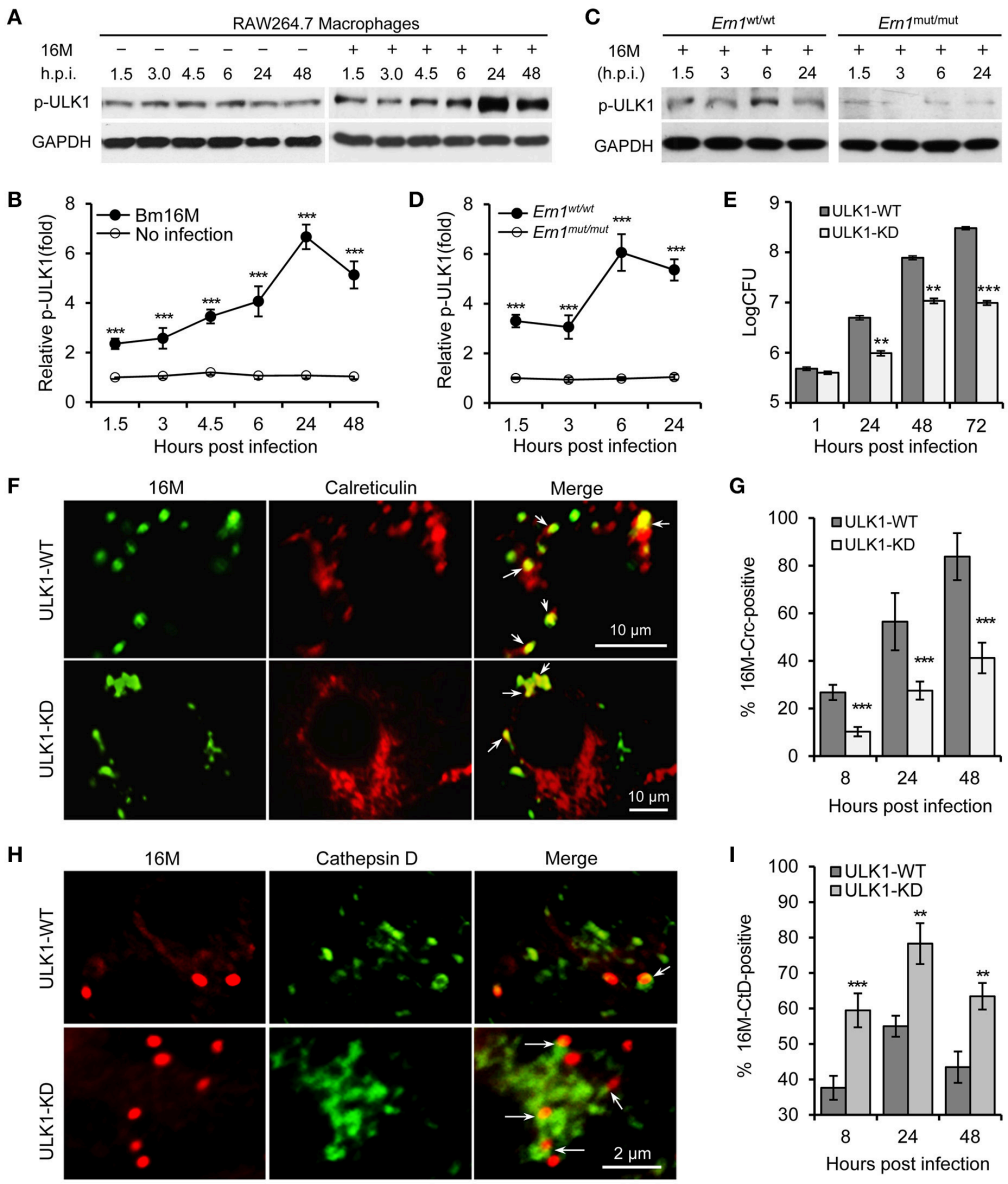
Host ULK1 activity contributes to *Brucella* intracellular trafficking and replication. Host cells were infected with Bm16M for the indicated lengths of times. Samples were lysed for Western blot or CFU assays, or fixed and processed for immunofluorescence with the indicated antibodies. **(A,B)** ULK1 activation during Bm16M infection **(A)** and quantification of the relative p-ULK1 level (mean of three blots for each time point) **(B)**. **(C,D)** Activation of host ULK1 is IRE1α-dependent during *Brucella* infection **(C)** and quantification of the relative p-ULK1 level **(D)**. **(E)** Bm16M intracellular replication was measured in ULK-depleted RAW264.7 macrophages. **(F,G)** Bm16M trafficking to and replication in calreticulin-positive compartments in control and ULK1-depleted host cells at 24 h.p.i. **(F)** and quantification of the Crc-positive BCVs in the infected cells at the indicated time points post-infection **(G)**. **(H,I)** Trafficking of Bm16M cells to lysosomes (marked by Cathepsin D) in control and ULK1-depleted RAW264.7 macrophages **(H)** and quantification of BCVs in these infected cells at the indicated time points post-infection **(I)**. Crc, calreticulin; CtD, cathepsin D. Data represent means ± *SD* from three independent experiments with triplicate wells examined for each treatment. ^**^p < 0.01 and ^***^p < 0.001, respectively.

Our findings in the insect cells implicated a role for ER-associated autophagy in conferring susceptibility to Bm16M intracellular parasitism (Figure S1). We therefore investigated whether kinase activity of ULK1 (Atg1), one of the main component in the autophagy initiation complex (Mizushima, 2010), played a role in Bm16M infection of mammalian cells. To test this possibility, we infected *Ern1*^wt/wt^ and *Ern1*^mut/mut^ BMDMs with Bm16M and monitored phosphorylation level of ULK1 during a time course of 48 h of infection. We also performed gentamicin protection assays and immunofluorescence microscopy assays to analyze Bm16M intracellular replication and trafficking in these BMDMs. We found that *Ern1*^wt/wt^ and *Ern1*^mut/mut^ BMDMs cultivated under low nutrient conditions displayed similar levels of ULK1 phosphorylation on Ser555 (Egan et al., 2011a,b; Kim et al., 2011), an event associated with the activation of this protein (Figure S3A). However, Bm16M infection enhanced the phosphorylation of ULK1 on Ser555 (Figures 4A–D). Moreover, the amount of phosphorylation of this residue was lower in uninfected RAW264.7 macrophages (Figures 4A,B) or in *Ern1*^mut/mut^ BMDMs infected with the pathogen (Figures 4C,D). These data indicated that host IRE1α activity controlled Bm16M-induced, but not nutrient deficiency-associated, ULK1 activation. Consistent with these findings was the observation that RAW264.7 macrophages that had been depleted of ULK1 proteins via lentiviral mediated gene silencing (Chaki et al., 2013; Pandey et al., 2017) reduced Bm16M intracellular parasitism (Figure 4E). In these cells, Bm16M trafficking to calreticulin-positive compartments was also reduced (compared to controls) (Figures 4F,G); however, the pathogen displayed enhanced trafficking to LAMP1-positive (Figures S3B,C), and cathepsin D-positive (Figures 4H,I) compartments. In addition, ULK1 co-localized with BCVs following infection (Figures S3D,E). These findings indicate that *Brucella* infection activation of host ULK1 is IRE1α dependent and promotes the pathogen intracellular trafficking and replication.

### IRE1α-mediated autophagy protein activities contribute to Bm16M intracellular parasitism

**Figure 5:**
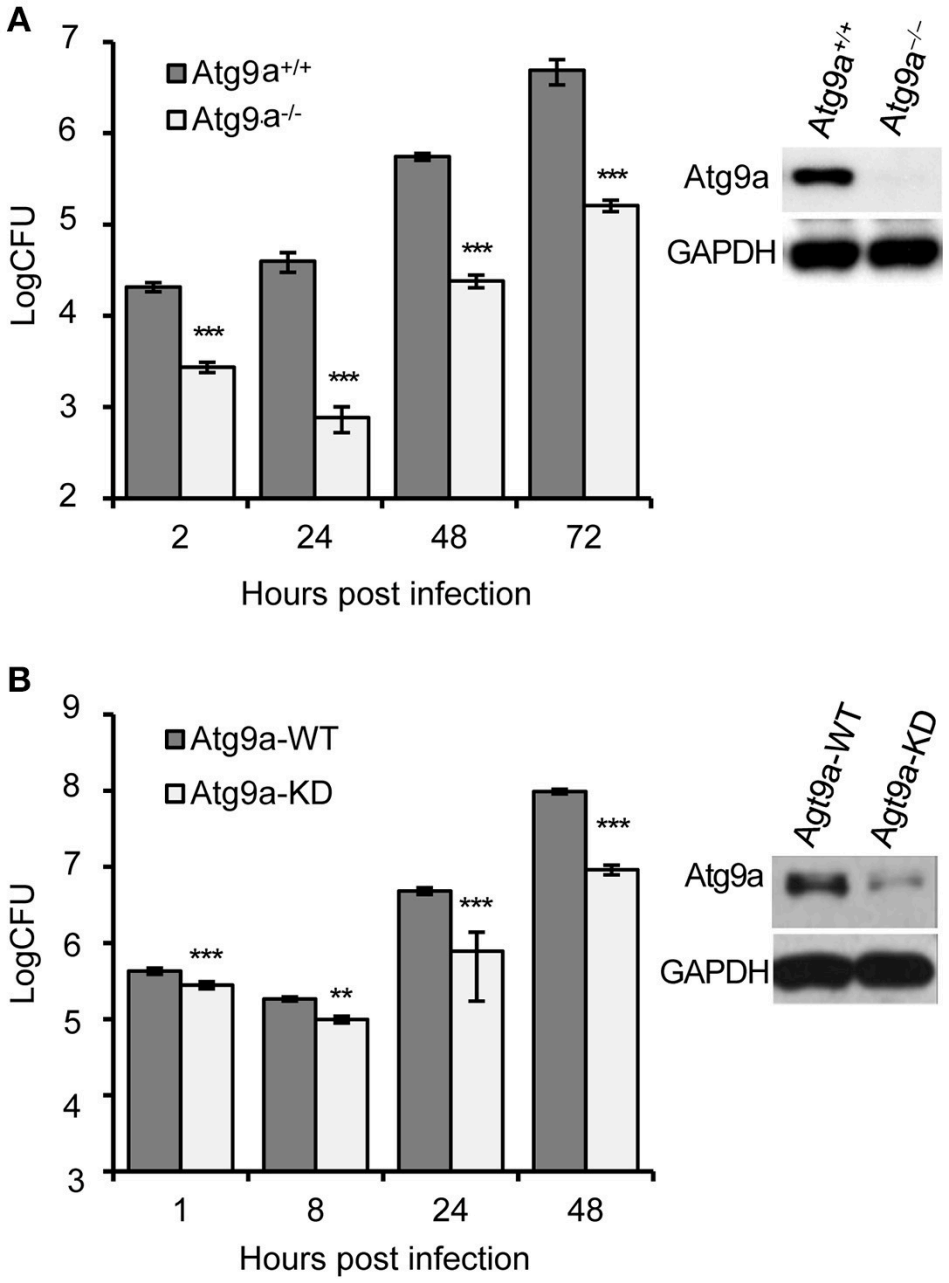
Host Atg9a supports Bm16M intracellular replication. **(A)** Bm16M intracellular replication in Atg9a^−/−^ MEF cells (upper right inset: Western blot demonstration of the absence of the Atg9a protein in the cells). **(B)** Bm16M intracellular replication was measured in Atg9a-depleted RAW264.7 macrophages (upper right inset: depletion of Atg9a in the cells detected by Western blot). Data represent means ± *SD* from three independent experiments with triplicate wells examined for each treatment. ^**^p < 0.01 and ^***^p < 0.001, respectively.

**Figure 6:**
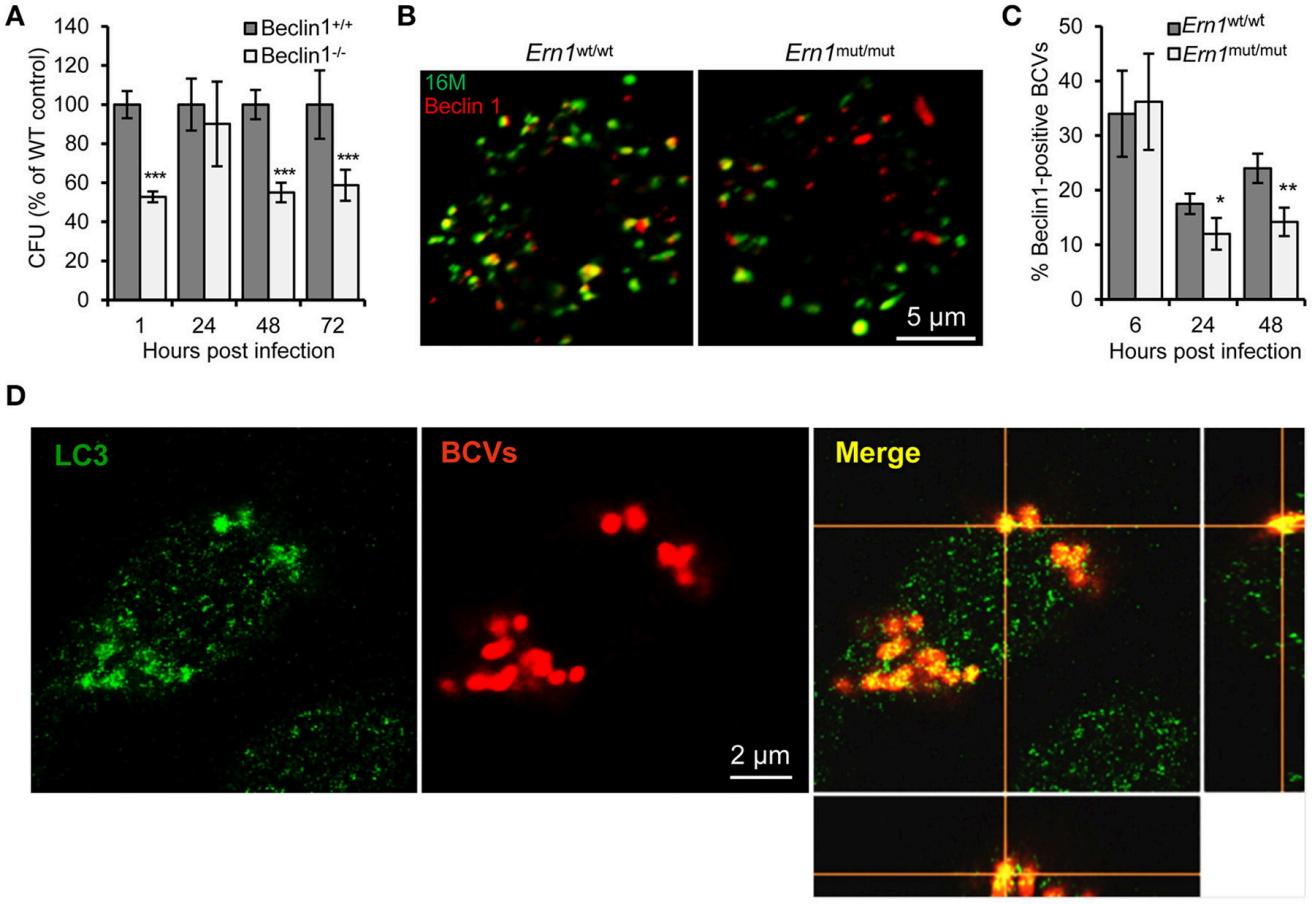
Host Atg proteins Beclin 1 and LC3 contribute to Bm16M intracellular parasitism. Host cells were infected with Bm16M. At the indicated h.p.i., the infected cells were lysed for CFU assays or fixed and processed for immunofluorescence with the indicated antibodies. **(A)** Bm16M infection of BMDMs in host cells in which Beclin 1 was ablated. **(B,C)** Bm16M trafficking to Beclin 1-positive compartments in *Ern1*^wt/wt^ and *Ern1*^mut/mut^ BMDMs at 48 h.p.i. **(B)** and quantification of Beclin 1-positive BCVs in the infected cells at the indicated time points post-infection **(C)**. **(D)** Accumulation of host LC3 near BCVs during Bm16M intracellular replication (24 h.p.i.) in RAW264.7 macrophages. Sections of the merged panels (right and bottom) showing the tight contact of Bm16M and LC3. Data represent means ± *SD* from three independent experiments with triplicate wells examined for each treatment. ^*^p < 0.05, ^**^p < 0.01, and ^***^p < 0.001, respectively.

To test the hypothesis that Atg9a activity conferred susceptibility to Bm16M infection, we examined Bm16M replication in Atg9a^−/−^ MEF cells (Saitoh et al., 2009; Figure 5A), as well as in RAW264.7 macrophages depleted of the protein via lentiviral mediated gene silencing (Figure 5B). We found that these Atg9a-deleted or -depleted cells had lower levels of intracellular Bm16M than WT controls (Figure 5). To test the hypothesis that Beclin 1 activity confers susceptibility to Bm16M infection, we examined the replication of the pathogen in Beclin1^−/−^ BMDMs via gentamicin protection and immunofluorescence microscopy assays. We found that Beclin 1-deficient cells supported lower levels of Bm16M infection than controls (Figure 6A). Moreover, during a time course (48 h) of Bm16M infection, less Beclin 1-positive BCVs were found in the *Ern1*^mut/mut^ BMDMs, indicating that Beclin 1 recruitment by BCVs is in an IRE1α-dependent fashion (Figures 6B,C; Figure S4A). These data suggest that IRE1α-mediated Beclin 1 association with BCVs promotes the intracellular trafficking and replication of the pathogen. Finally, to investigate a role of IRE1α-mediated LC3-associataed autophagy in Bm16M intracellular parasitism, we examined the effect of IRE1α activity on LC3 processing. We found that IRE1α-deficiency impaired LC3 processing in Bm16M infected BMDMs (Figures S4B,C). Moreover, endogenous LC3 surrounding or accumulating near BCVs was observed during pathogen infection of host cells (Figure 6D; Figure S4D; Movie S1), thereby suggesting that host LC3 and IRE1α-mediated LC3 processing may contribute to the intracellular lifestyle of Bm16M. Taken together, the data suggest a mechanism whereby the activity of a novel IRE1α-ULK1 signaling axis promotes Bm16M intracellular parasitism.

### IRE1α-mediated AMPKα activity supports Bm16M intracellular replication

**Figure 7:**
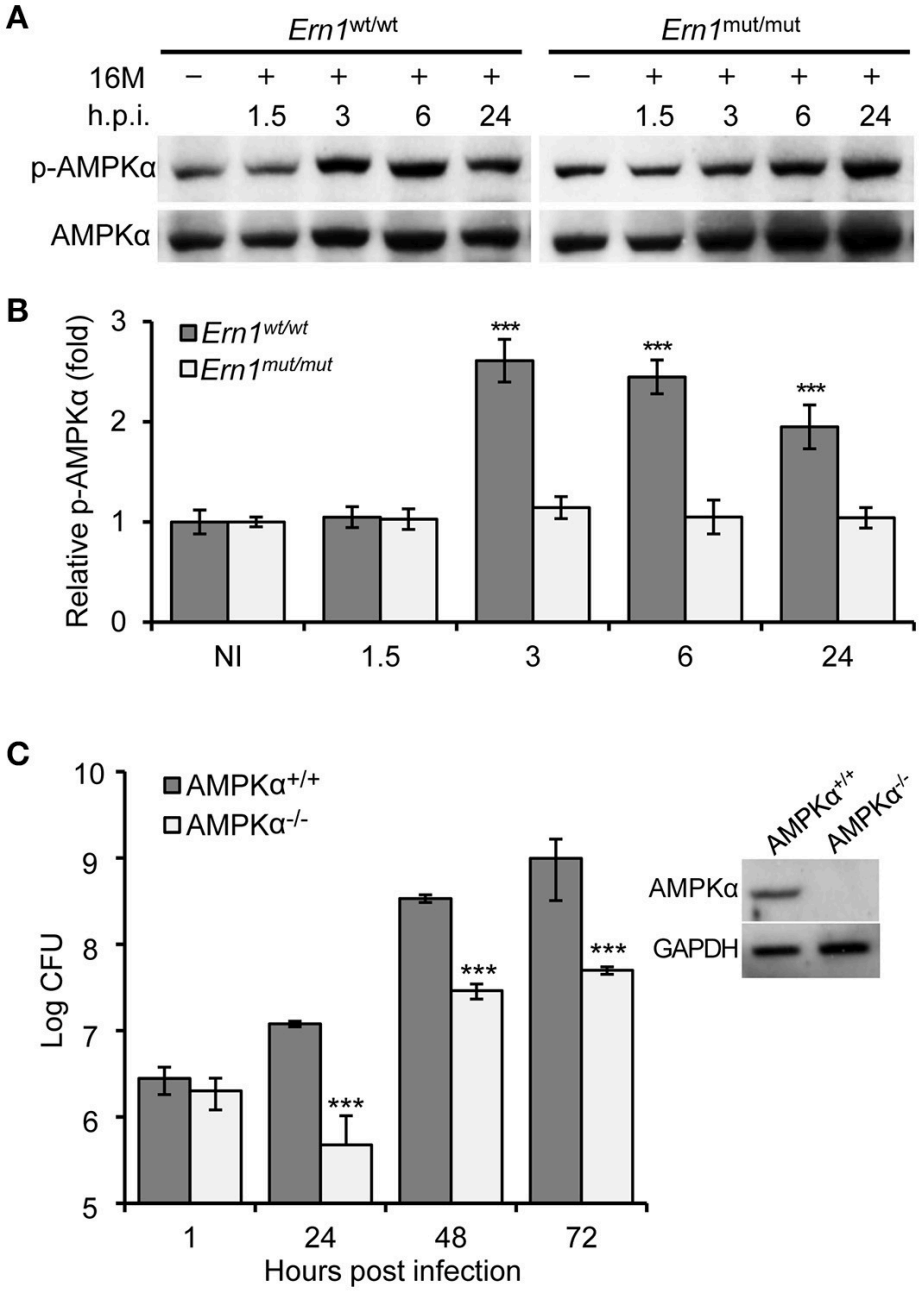
Host AMPKα activation during Bm16M infection. The indicated host cells were infected with Bm16M. At the indicated time points post-infection, infected cells were lysed for Western blot or CFU analysis. **(A,B)** Activation of host AMPKα during Bm16M infection of control and *Ern1*^mut/mut^ BMDMs **(A)** and quantification of the relative p-AMPKα level (mean of three blots for each time point) **(B)**. NI, no infection. **(C)** Bm16M intracellular replication in BMDMs derived from control or conditional AMPKα KO mice. The inset demonstrates deficiency of host AMPKα in BMDMs from conditional KO mice. Data represent means ± *SD* from three independent experiments with triplicate wells examined for each treatment. ^***^p < 0.001.

Liu and colleagues recently showed that IRE1α activity promotes *B. abortus* growth by activating AMPKα, which suppresses NADPH-derived ROS production and limits infection (Liu et al., 2016). During starvation, activated AMPK regulates autophagy through direct phosphorylation of ULK1 (Kim et al., 2011). To test whether Bm16M-induced activation of ULK1 could also occur through the activation of AMPKα, we examined the phosphorylation AMPKα in the *Ern1*^wt/wt^ or *Ern1*^mut/mut^ BMDMs during a time course of Bm16M infection. We found that AMPKα displayed enhanced phosphorylated (Thr172) in infected *Ern1*^wt/wt^ BMDMs (compared to *Ern1*^mut/mut^ controls) (Figures 7A,B), and moreover, that AMPKα^−/−^ cells (Pandey et al., 2017) reduced Bm16M intracellular replication (Figure 7C). Our findings that AMPKα was phosphorylated in an IRE1α-dependent fashion in Bm16M infected cells, and that mouse BMDMs harboring deletions in AMPKα showed lower levels of infection, are consistent with these findings (Liu et al., 2016). These data therefore suggest that Bm16M activation of host autophagy components may also be partially achieved through the activity a signaling pathway that couples IRE1α and AMPKα.

## Discussion

This report demonstrates that disruption of IRE1α activity in host cells reduces *Brucella* intracellular replication by initiating a signaling cascade that regulates the maturation of BCVs. We also showed that depletion or ablation of host IRE1α, BAK/BAX, ASK1, JNK, ULK1, Beclin 1, ATG9a, or AMPKα results in reduced intracellular replication of Bm16M. Our findings support the hypothesis that the activity of an IRE1α-ULK1 signaling axis and IREα-dependent autophagy in host cells contributes to conferring susceptibility to the intracellular replication of Bm16M. However, host cells harboring deficiencies in some of these proteins, e.g., ULK1, do not display dramatically alterations in pathogen entry, thereby suggesting that this axis may play a diminished role in regulating this process. Future work will investigate this possibility.

A few previous reports have suggested that *Brucella* infection is not associated with the induction of host cell UPR (Starr et al., 2012) and IRE1α activity does not contribute to *Brucella* intracellular parasitism of host cells (de Jong et al., 2013). However, other reports have demonstrated UPR induction by infection with *Brucella* spp. (de Jong et al., 2013; Smith et al., 2013; Taguchi et al., 2015; Wang et al., 2016) and the requirement of IRE1α activity for the intracellular replication of the pathogen (Qin et al., 2008; Smith et al., 2013; Taguchi et al., 2015). Starr and colleagues previously demonstrated that ULK1 and Beclin 1 are dispensable for *B. aborts* intracellular replication; siRNA-mediated depletion of these components in host (HeLa) cells did not affect replication, but were required for the formation of aBCVs that are involved in the release of replicative bacteria and pathogen reinfection of host cells. Depletion of these two Atg proteins significantly impairs these processes. Starr et al. also reported that membranes of BCVs do not accumulate host LC3 at any stage of infection in host cells (Starr et al., 2012). Our data demonstrate that *Brucella* intracellular infection was impaired in Beclin 1 KO BMDMs and in ULK1-depleted RAW264.7 macrophages. We also observed endogenous LC3 puncta surrounding or accumulating near BCVs (at 24 h.p.i.) in these cells. It has been noted that *Brucella* does not behave exactly the same in macrophage and non-phagocytic cells (e.g., HeLa cells) (Arenas et al., 2000). The differences in the cell models used, the levels and the maintenance of IRE1α kinase inhibition or target protein-depletion achieved, or the experimental approaches employed in these studies, may account for the observed discrepancies.

The roles of IRE1α-dependent autophagy and autophagy-associated proteins in regulating *Brucella* spp. infection of host cells remain to be characterized. *B. melitensis* infection of RAW264.7 macrophages induces the conversion of LC3-I to LC3-II (LC3-PE) and autophagic flux, which favors intracellular replication of the bacteria (Guo et al., 2012). Our observation that Bm16M infection of RAW264.7 macrophages increased the ratio of LC3-II to LC3-I is consistent with these findings. Endogenous LC3 puncta are also observed to surround or accumulate near BCVs during bacterial intracellular replication (at 24 h.p.i.) in RAW264.7 macrophages (Figures 6D; Figure S4D; Movie S1). In mitaphagy, a process of mitochondrial degradation by autophagy, structures containing upstream Atg proteins, including ULK1, Atg14, DFCP1, WIPI1, and Atg16L1, can associate with depolarized mitochondria in the presence or absence of LC3. In starvation-induced canonical autophagy, Atg9A structures are also recruited to the damaged mitochondria as well as to sites of autophagosome formation. In Parkin (a Parkinson-disease-associated ubiquitin ligase that can trigger depolarized-mitochondrion mitophagy) -mediated mitophagy, the structures containing Atg9A and the ULK1-complex are independently recruited to depolarized mitochondria and both are required for further recruitment of downstream Atg proteins, except LC3. However, LC3-II is important for efficient incorporation of damaged mitochondria into the autophagosome at a later stage (Itakura et al., 2012). Like mitophagy, the presence of LC3 near BCVs may also promote BCV fusion to generate aBCVs. Host Atg9a and WIPI1 are required for the generation of replicative BCVs and intracellular replication (Taguchi et al., 2015). ULK1 and Beclin 1 are also observed to contribute to bacterial intracellular growth in RAW264.7 or BMDM cells. The contribution of Atg proteins, including ULK1 and Beclin 1, to *Brucella* intracellular parasitism remains to be further clarified.

**Figure 8:**
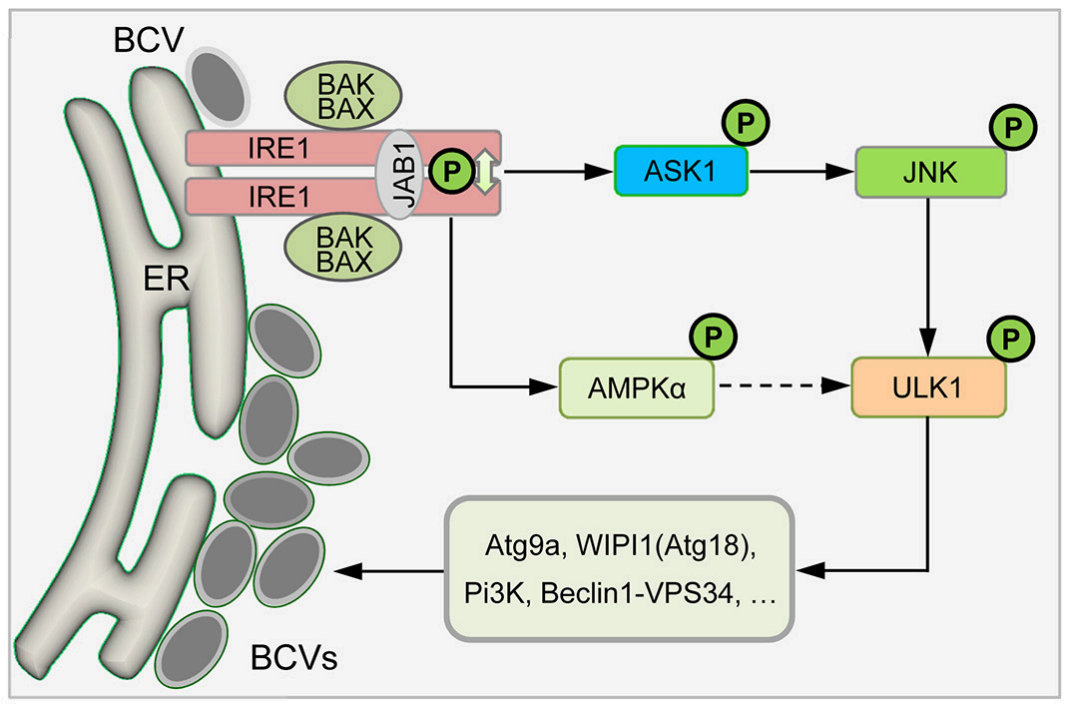
Proposed model describing host IRE1α-ULK1 signaling axis mediating *Brucella* intracellular lifestyle. Internalized *Brucella* activates host UPR sensor IRE1α that drives the activation of IRE1α-associated kinases, including ASK1, JNK, and/or AMPKα. The activities of these IRE1α-associated signaling proteins drive the activation or assembly of downstream proteins, including ULK1, Beclin 1 and Atg9a, as well as drive the remodeling of cellular membranes to support *Brucella* intracellular parasitism.

Our data suggest a step-wise mechanism whereby *Brucella* subverts a novel host IRE1α-ULK1 signaling axis to promote the intracellular trafficking and replication of the pathogen (Figure 8). First, *Brucella* entry into host cells and/or trafficking to the ER either directly or indirectly induces host cell UPR and activates IRE1α signaling (Smith et al., 2013; Taguchi et al., 2015; Liu et al., 2016; Wang et al., 2016). Second, activated IRE1α drives the activation of IRE1α-associated kinases, including ASK1 and JNK (Hetz et al., 2006; Ogata et al., 2006). Third, the activities of these IRE1α-associated signaling proteins drive the activation or assembly of downstream proteins, including ULK1, Atg9a, WIPI1, and Beclin 1, key components of the host autophagy machinery (Ogata et al., 2006; Wei et al., 2008; Egan et al., 2011b; Kim et al., 2011). Finally, the activities of these autophagy proteins drive the remodeling of cellular membranes to support the development of an intracellular replicative niche that supports the intracellular parasitism of the pathogen.

Evidence from recent reports supports our working model. Smith and colleagues elegantly demonstrated that host UPR activity is critical for the intracellular replication of *Brucella*, and moreover, that TcpB and potentially other factors, promote host UPR to enable the intracellular replication of the agent (Smith et al., 2013). These data are consistent with our findings that IRE1α activity regulates the intracellular trafficking and replication of the pathogen. We previously demonstrated that among the host UPR sensors, only IRE1α, but not PERK and ATF6, contributes to *Brucella* intracellular parasitism (Qin et al., 2008). Similar to these findings, Taguchi et al. recently showed that *B. abortus* infection activates IRE1α, but not PERK and ATF6, and that prevention of IRE1α activation by yip1A inhibits the development of ER-derived BCVs (Taguchi et al., 2015). The observation that reduction of intracellular bacteria in Atg9a KO and KD mammalian cells and in Atg18 (WIPI1) KD mammalian (Taguchi et al., 2015) and macrophage-like S2 cells, as well as both ULK1 and Beclin 1 association with BCVs (this study), support this hypothesis. In addition, it has recently been shown that ULK1 regulates ER-to-Golgi trafficking to maintain cellular homeostasis (Joo et al., 2016). The observation that proteins that control the activities of ER-exit sites also control *Brucella* replication (Taguchi et al., 2015) further supports our working model. The previous findings, and those reported here, demonstrating that host autophagy components regulate BCV biogenesis and subcellular trafficking are consistent with the proposed model. IRE1α-dependent activation of AMPKα contributes to *B. abortus* (Liu et al., 2016) and *B. melitensis* (this study) intracellular replication. However, during *Brucella* intracellular replication, activated AMPKα contribution to activation of ULK1 and assembly of downstream proteins that promote *Brucella* intracellular parasitism remains to be further clarified. Finally, this report leaves open intriguing questions about how IRE1α is activated at early stages of infection. In addition, the effect that molecules in the XBP1-independent pathway have on whole animal infections remains to be interrogated. Future work in our labs will be directed toward addressing these questions.

## Author contributions

Q-MQ, PD, and AP conceived and designed the experiments. Q-MQ, AP, FL, AC, LC, XF, H-QF, M-ZZ performed the experiments. Q-MQ, PD, TF, and AP analyzed the data. Q-MQ, PD, TF, TI, and AR-F contributed reagents, materials, and analysis tools. Q-MQ and PD supervised the work and wrote the paper.

### Conflict of interest statement

The authors declare that the research was conducted in the absence of any commercial or financial relationships that could be construed as a potential conflict of interest.

## Supplementary material

Figure S1 Depletion of components of the IRE1α signaling cascade in a heterologous host decreased susceptibility to *Brucella melitensis* 16M (Bm16M) infection. *Drosophila melanogaster* S2 cells were depleted of the indicated target genes using RNAi approaches and then infected with wild-type (WT) Bm16M-GFP for the indicated lengths of time. Samples were lysed for CFU assays or fixed and processed for immunofluorescence microscopy. Vps34 and Pi3ks were used as negative controls since they support *B. abortus* (S2308) and Bm16 host infection. **(A)** Bm16M burden in *Drosophila* S2 cells depleted of the indicated host components in the IRE1α signaling cascade at 1 or 72 h.p.i. (hours post infection). **(B)** Representative images showing intracellular Bm16M in S2 cells following depletion of the indicated UPR-related components at 72 h.p.i. Data represent means ± *SD* from three independent experiments with triplicate wells examined for each treatment. ^***^*p* < 0.001 compared to the control.

Figure S2 Host JNK1/2 is active in Bm16M-infected JNK1^+/+^ and JNK1^−/−^ MEFs. **(A)** Activation of host JNK1/2 during Bm16M infection of JNK1^+/+^ and JNK1^−/−^ MEFs. **(B)** Quantification of the relative p-JNK1 and p-JNK2 levels. NI, no infection. Rp-JNK1 and Rp-JNK2: relative phosphorylation levels of p-JNK1 and p-JNK2, respectively.

Figure S3 Autophagy initiating Kinase ULK1 mediates *Brucella* intracellular trafficking and replication. Host cells were infected with Bm16M for the indicated lengths of time. Samples were lysed for Western blot, or CFU assays, or fixed and processed for immunofluorescence with the indicated antibodies. **(A)** IRE1α is dispensable for ULK1 phosphorylation on Ser555 under low nutrition conditions. CT, culture time (hrs). **(B)** Bm16M trafficking to host lysosome compartments (marked by LAMP1) in host cells depleted of ULK1. **(C)** Quantification of Bm16M trafficking to the lysosomes in control or ULK1-depleted RAW264.7 macrophages at the indicated time points post-infection. **(D)** Co-localization of BCVs and host ULK1 during infection. **(E)** Quantification of BCVs decorated with host ULK1 protein at the indicated time points post infection. Data represent means ± *SD* from three independent experiments with triplicate wells examined for each treatment. ^***^*p* < 0.001.

Figure S4 Autophagy components contribute to *Brucella* intracellular parasitism. **(A)** Differential co-localization patterns of host Beclin 1 (BECN) and BCVs in control and *Ern1*^mut/mut^ BMDMs at the indicated time points post-infection. **(B,C)** Host LC3 conversion from LC3-I to LC3-II during Bm16M intracellular replication in *Ern1*^*wt*/*wt*^ and *Ern1*^*mut*/*mut*^ BMDMs **(B)** and quantification of the ratio of blot LC3-II/LC3-I at the indicated time points post-infection **(C)**. **(D)** A representative image demonstrating accumulation of host LC3 (green) near BCVs (red) at 24 h.p.i. in three dimensions. ^**^*p* < 0.01 and ^***^*p* < 0.001, respectively.

Movie S1 Accumulation of host LC3 near BCVs during *Brucella* intracellular replication. Stacked images (at 24 h post-infection) are shown.
