# Supplementary figures and images for: Activation of Host IRE1α-Dependent Signaling Axis Contributes the Intracellular Parasitism of Brucella melitensis
Source: Front Cell Infect Microbiol. 2018 Apr 20;8:103. doi: 10.3389/fcimb.2018.00103 (PMC5919948; doi:10.3389/fcimb.2018.00103)

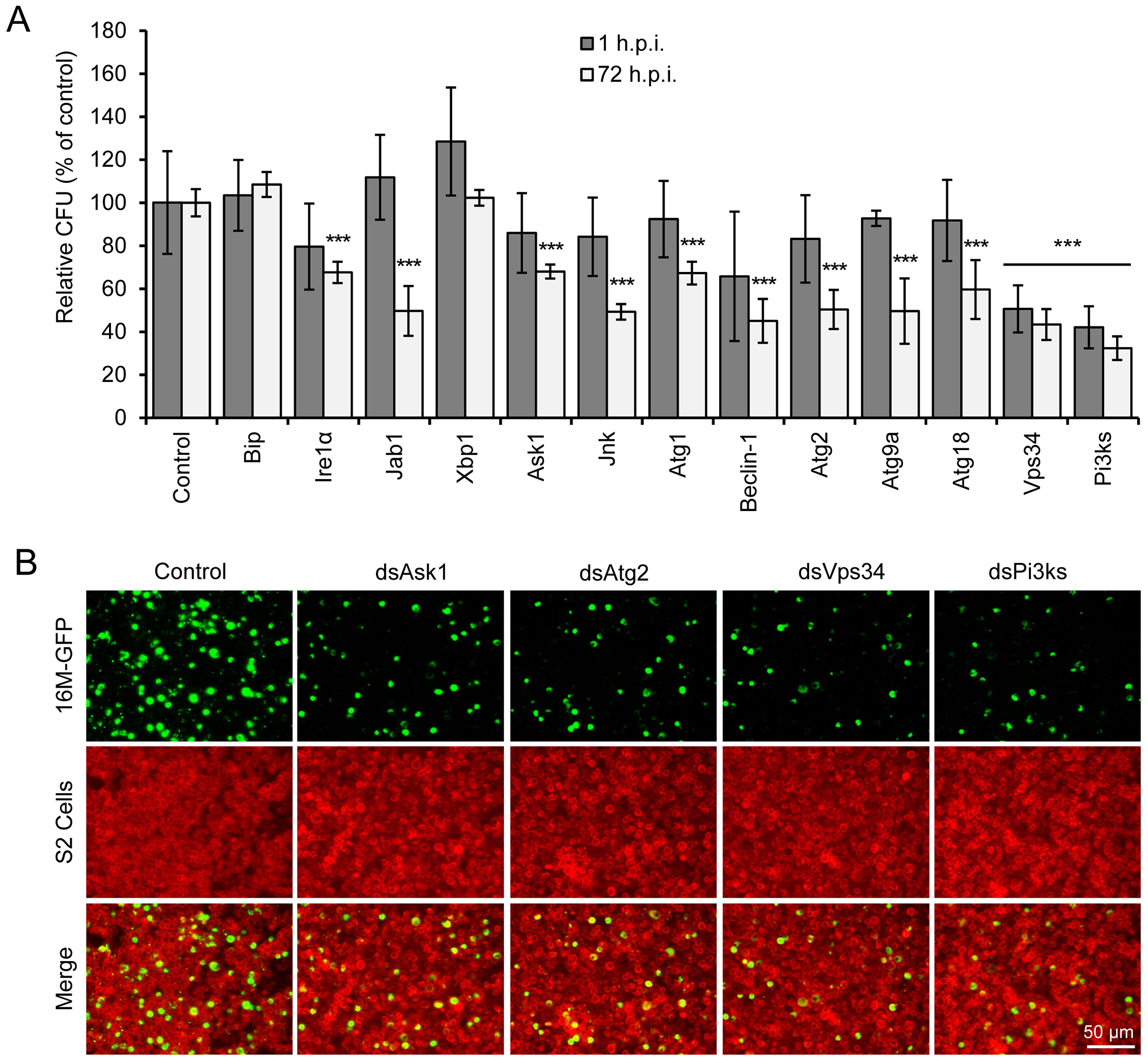

Supplement: Figure S1 — Depletion of components of the IRE1α signaling cascade in a heterologous host decreased susceptibility to Brucella melitensis 16M (Bm16M) infection. Drosophila melanogaster S2 cells were depleted of the indicated target genes using RNAi approaches and then infected with wild-type (WT) Bm16M-GFP for the indicated lengths of time. Samples were lysed for CFU assays or fixed and processed for immunofluorescence microscopy. Vps34 and Pi3ks were used as negative controls since they support B. abortus (S2308) and Bm16 host infection. (A) Bm16M burden in Drosophila S2 cells depleted of the indicated host components in the IRE1α signaling cascade at 1 or 72 h.p.i. (hours post infection). (B) Representative images showing intracellular Bm16M in S2 cells following depletion of the indicated UPR-related components at 72 h.p.i. Data represent means ± SD from three independent experiments with triplicate wells examined for each treatment. ***p < 0.001 compared to the control. [file Image1.TIF]

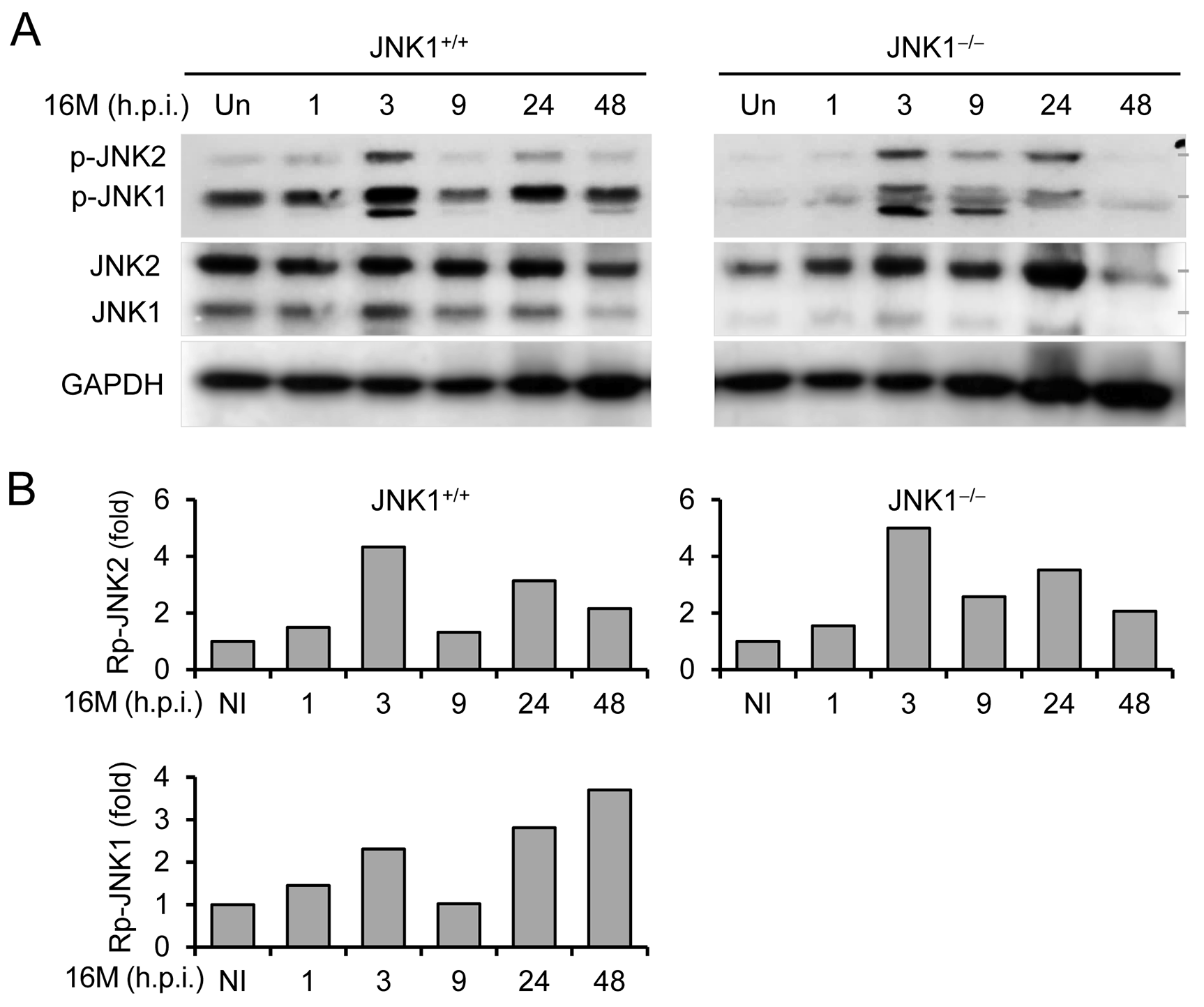

Supplement: Figure S2 — Host JNK1/2 is active in Bm16M-infected JNK1+/+ and JNK1−/− MEFs. (A) Activation of host JNK1/2 during Bm16M infection of JNK1+/+ and JNK1−/− MEFs. (B) Quantification of the relative p-JNK1 and p-JNK2 levels. NI, no infection. Rp-JNK1 and Rp-JNK2: relative phosphorylation levels of p-JNK1 and p-JNK2, respectively. [file Image2.TIF]

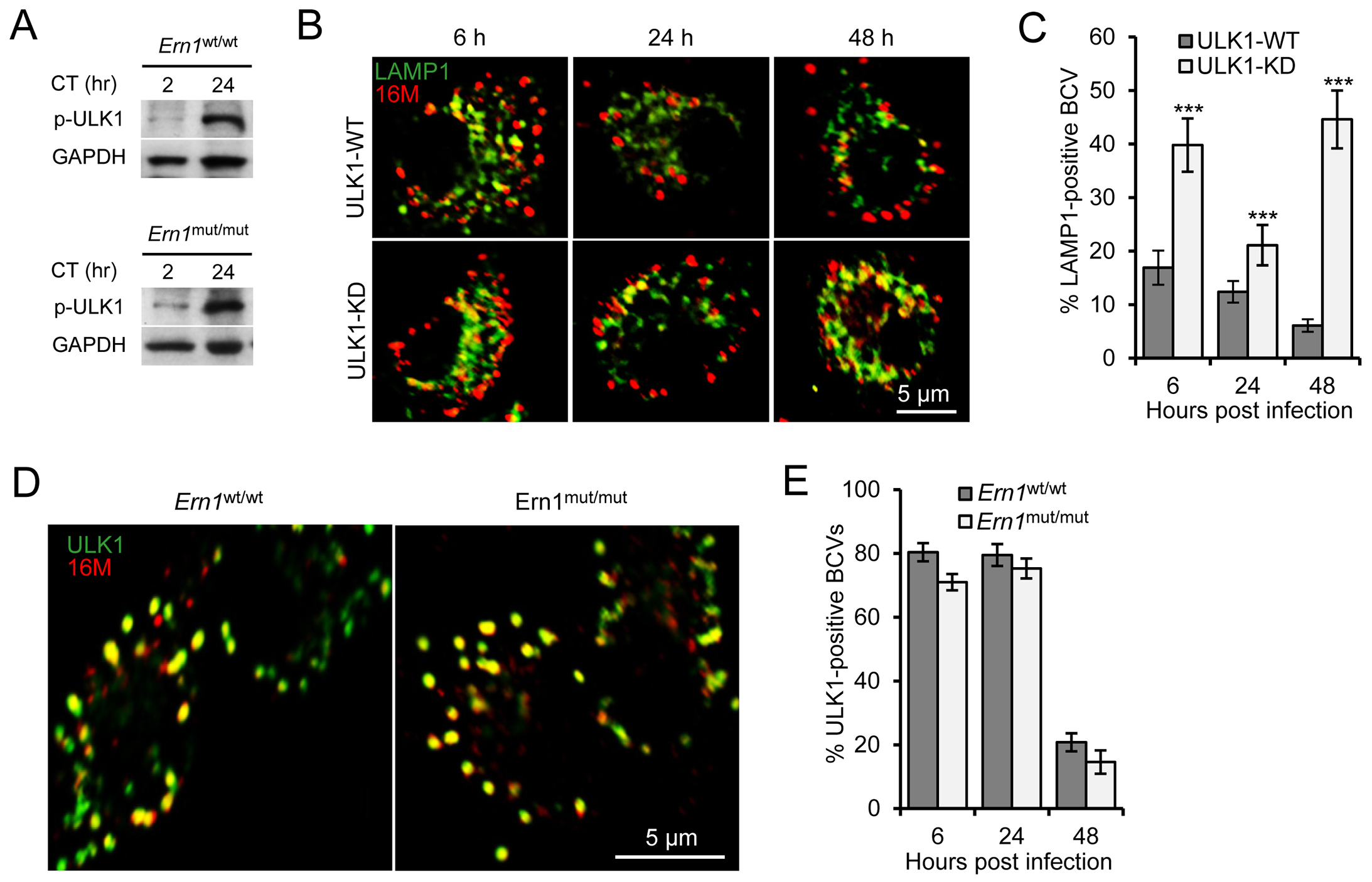

Supplement: Figure S3 — Autophagy initiating Kinase ULK1 mediates Brucella intracellular trafficking and replication. Host cells were infected with Bm16M for the indicated lengths of time. Samples were lysed for Western blot, or CFU assays, or fixed and processed for immunofluorescence with the indicated antibodies. (A) IRE1α is dispensable for ULK1 phosphorylation on Ser555 under low nutrition conditions. CT, culture time (hrs). (B) Bm16M trafficking to host lysosome compartments (marked by LAMP1) in host cells depleted of ULK1. (C) Quantification of Bm16M trafficking to the lysosomes in control or ULK1-depleted RAW264.7 macrophages at the indicated time points post-infection. (D) Co-localization of BCVs and host ULK1 during infection. (E) Quantification of BCVs decorated with host ULK1 protein at the indicated time points post infection. Data represent means ± SD from three independent experiments with triplicate wells examined for each treatment. ***p < 0.001. [file Image3.TIF]

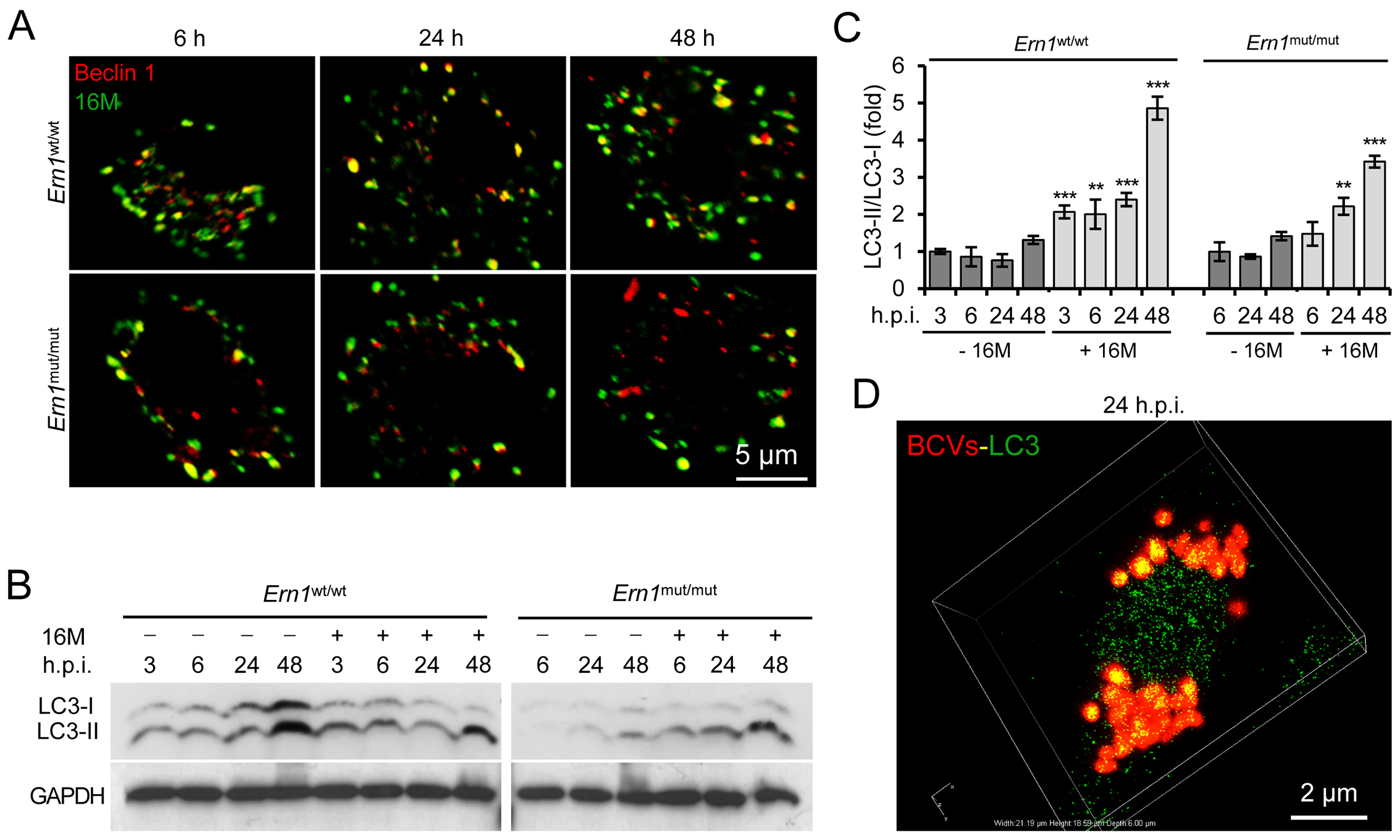

Supplement: Figure S4 — Autophagy components contribute to Brucella intracellular parasitism. (A) Differential co-localization patterns of host Beclin 1 (BECN) and BCVs in control and Ern1mut/mut BMDMs at the indicated time points post-infection. (B,C) Host LC3 conversion from LC3-I to LC3-II during Bm16M intracellular replication in Ern1wt/wt and Ern1mut/mut BMDMs (B) and quantification of the ratio of blot LC3-II/LC3-I at the indicated time points post-infection (C). (D) A representative image demonstrating accumulation of host LC3 (green) near BCVs (red) at 24 h.p.i. in three dimensions. **p < 0.01 and ***p < 0.001, respectively. [file Image4.TIF]
